# Risk of adverse swallowing events and choking during deworming for preschool-aged children

**DOI:** 10.1371/journal.pntd.0006578

**Published:** 2018-06-22

**Authors:** James Wyatt Kernell, Rosalie V. DePaola, Alec M. Maglione, Lacey N. Ahern, Naomi G. Penney, David G. Addiss

**Affiliations:** 1 Eck Institute for Global Health, University of Notre Dame, Notre Dame, Indiana, United States of America; 2 Children Without Worms, Task Force for Global Health, Decatur, Georgia, United States of America; 3 Focus Area for Compassion and Ethics, Task Force for Global Health, Decatur, Georgia, United States of America; Swiss Tropical and Public Health Institute, SWITZERLAND

## Abstract

**Background:**

In areas where the prevalence of soil-transmitted helminthiasis (STH) is >20%, the World Health Organization (WHO) recommends that deworming medication be given periodically to preschool-age children. To reduce risk of choking-related deaths in children <3 years old, WHO recommends that deworming tablets be crushed and given with water. Little is known about how widely this is practiced or its effectiveness.

**Methodology and principal findings:**

Albendazole distributions for STH were observed for children 1–4 years old in 65 sites in India and Haiti. Information was recorded on child demographics; child demeanor immediately *before*, as well as struggling or resistance *during* albendazole administration; tablet form (i.e., crushed or not); and adverse swallowing events (ASEs), including choking, spitting; coughing; gagging; vomiting; and expelling a crushed tablet in a “cloud” of powder. Of 1677 children observed, 248 (14.8%) had one or more ASEs. ASE risk was 3.6% with whole tablets, 25.4% with crushed tablets, and 34.6% when crushed tablets were mixed with water. In multivariate analysis, ASE risk was significantly associated with children 1 year (OR 2.7) or 2 years (OR 2.9) of age; male gender (OR 1.6); non-content child demeanor (fearful, fussy, or combative) before albendazole administration (OR 4.3); child struggling when given albendazole (OR 2.1); and giving water, either after the tablet or mixed with it (OR 5.8). Eighteen (1.1%) children choked, none fatally; 17 choking incidents occurred with crushed tablets. In a multivariate analysis that controlled for distribution site, the only significant risk factor for choking was non-content demeanor (OR 20.6).

**Conclusions and significance:**

Deworming-related choking deaths in young children are preventable. In our sample, risk of choking could have been reduced by 79.5% if deworming tablets were not given to young children who were fussy, fearful, or combative or who struggled to resist tablet administration, with only an 18.4% reduction in drug coverage.

## Author summary

To improve the health of young children with intestinal worm infections, the World Health Organization recommends that they periodically receive deworming medication. The medicines are safe and effective but young children occasionally choke on the chewable tablets. Rarely, this results in death. Consequently, the World Health Organization recommends that deworming tablets be crushed and given with water. To learn more about the risk of choking, we observed 1677 children 1–4 years of age being dewormed in 65 sites in India and Haiti. Of these, 18 children (1.1%) choked, none fatally. The most important factor associated with choking was giving a tablet to a child who was fussy, fearful or combative. Risk of choking could have been reduced by 79.5% if deworming was not forced on young children who were fussy, fearful, or combative or who resisted taking deworming medicine. Choking-related deaths in young children are preventable. Simple measures can reduce risk of choking and they should be more widely practiced by deworming programs for young children.

## Introduction

Soil-transmitted helminthiasis (STH) is a group of parasitic diseases caused by the nematode worms *Ascaris lumbricoides* (roundworm), *Trichuris trichiura* (whipworm), *Ancylostoma duodenale* and *Necator americanus* (hookworm). The worms are transmitted to humans through fecal contamination of soil, either through skin penetration by larvae or through ingestion of embryonated eggs [1]. Although infection often results in subtle and non-specific symptoms such as malaise, nausea, and abdominal pain, STH is also associated with anemia, wasting, and impaired cognitive development [1–3]. More than one billion persons are affected by STH worldwide [4].

When the World Health Organization (WHO) launched its initiative to eliminate STH as a public health problem in 2001 [5], mass deworming, known as preventive chemotherapy, was focused largely on school-age children using schools as convenient, low-cost drug distribution posts [6]. In recognition of the inadequately addressed but significant public health burden of STH in younger children [7], WHO broadened its program to also emphasize at-risk preschool-age children (1–4 years, i.e., 12–59 months), with the aim of regularly reaching 75% with preventive chemotherapy by 2020 [8]. In 2015, 269 million children 1–4 years of age were considered at risk of STH and in need of preventive chemotherapy; an estimated 130 million (48%) were treated [9]. Many of these children were treated during “child health days,” in which deworming was provided with vitamin A supplements and other interventions [10].

The benefits of mass deworming are well-documented [1,2,7,11], although population-level effects may be hard to measure when STH prevalence is low [12–14]. Single doses of albendazole or mebendazole are safe and effective for preventive chemotherapy [15,16]. In STH-endemic countries, these drugs are most commonly available as chewable tablets. While more appropriate for young children than non-chewable tablets, chewable tablets for STH have been implicated in choking deaths of young children. The frequency with which this occurs in the context of preventive chemotherapy for STH is apparently low. However, the actual number is unknown because of the low sensitivity of serious adverse event (SAE) surveillance and the absence of a global reporting system for SAEs in young children being treated for STH. These factors hinder the collection of crucial information on the circumstances of the drug administration and subsequent events, making it difficult to draw accurate inferences regarding causality and, most importantly, to prevent further deaths.

Indirect sources provide a crude estimate of the magnitude of the problem. In 2007, WHO reported four fatal cases of choking in Ethiopian children who were given albendazole for STH [17]. Deaths related to preventive chemotherapy continue to be reported in the media [18], although it is difficult to be certain of causality in many of these cases. Between 2011 and 2016, the Children Without Worms program [19] was alerted, on average, to at least one suspected choking-related fatality every year (D. Addiss, personal communication). Anecdotally, these cases often occur when the child, struggling to resist taking a whole tablet, aspirates and the tablet lodges in the trachea. While one death per year represents a small number relative to the number of children dewormed [9], any such death is tragic and unacceptable.

Between 2004 and 2007, WHO issued three statements or recommendations for administering deworming medicine to young children in mass treatment settings. In 2004, WHO suggested that “the tablets can be crushed between two spoons and given with a glass of water for children that have difficulties in swallowing the tablets” (p. 13) [20]. In a 2007 document, *Promoting Safety of Medicines for Children* [17], WHO recommended that “scored tablets should be broken into smaller pieces or crushed for administration to young children; older children should be encouraged to chew tablets of albendazole” (p. 11). Age was not specified and the method of crushing the tablet was not indicated. Finally, in a 2007 edition of a Partnership for Parasite Control (PPC) newsletter, *Action Against Worms* [21], WHO recommended that, “For children under 3 years of age, tablets should be broken and crushed between two spoons, then water added to help administer the tablets (p. 7).” The document highlighted the need for training, supervision, and not forcing the child to take the medicine, and it recommended that drug distributors be trained in the Heimlich maneuver. Little is known about the extent to which the WHO recommendations are actually practiced.

Vitamin Angels, a non-governmental organization (NGO), provides vitamin A supplements and albendazole, as well as training in their administration, to hundreds of NGOs in countries that are co-endemic for STH and vitamin A deficiency [22]. Vitamin Angels recommends that its partner NGOs crush albendazole tablets for all children less than 59 months of age, using a heavy bottle or other object to crush the tablets inside a folded piece of clean paper. The crushed tablet is then poured from the paper into the mouth of the child [23].

Vitamin Angels also recommends specific infection control measures to reduce transmission of respiratory and other pathogens, since these organisms are readily spread in health care settings [24]. They stipulate that the service provider (the person administering the tablet) avoid physical contact with the child, wash his or her hands between children, use a clean piece of paper to administer the crushed tablet to each child, and dispose of the paper after each use [23].

We observed distributions of albendazole for STH to children 1–4 years of age in India and Haiti. The objectives were to: 1) evaluate the extent to which service providers are aware of and practice WHO recommendations for deworming preschool-age children; 2) document the incidence of, and identify risk factors for, adverse swallowing events, including choking; and 3) assess whether the approach recommended by Vitamin Angels is associated with a) greater likelihood of delivering the correct dose and the WHO-recommended form of albendazole (i.e., crushed, rather than whole, tablets for children 1–2 years of age); b) decreased risk of adverse swallowing events; and c) improved infection control practices. To address the third objective, observations were conducted both at sites affiliated with Vitamin Angels and those that were not.

## Methods

The sample of children observed was a convenience sample, drawn from seven organizations in India and six in Haiti that were known to the national Vitamin Angels consultants. During May-June 2017, distributions of vitamin A supplements and albendazole were scheduled at 38 of 419 sites run by these organizations in India and 27 of 60 such sites in Haiti. All 65 of these sites were included in the sample. In India, the distribution sites observed were located in Odisha, Maharashtra, West Bengal, and Tamil Nadu. In Haiti, observations were conducted in the Sud and Ouest Departments. All distribution sites were administered by local NGOs.

Just before each session, data were collected about the specific site visited; the organization sponsoring the drug distribution; and characteristics of the facility where the drug distribution took place (e.g., whether a school, clinic, other facility, or outdoors). For each child receiving albendazole for deworming, evaluators observed and recorded information on child age and gender; demeanor (content, fussy, fearful, or combative) immediately before drug delivery; whether the child struggled to avoid or resisted taking the tablet; drug dose (200 mg or 400 mg) and form (i.e., whether the tablet was crushed); whether water was given to the child; adverse swallowing events; who (if anyone) supported the child’s head during drug delivery; and whether the service provider touched the child. An adverse swallowing event was defined as choking with airflow (child able to vocalize while choking); choking with no airflow (child in distress and unable to vocalize or breathe); spitting; coughing; gagging; vomiting; or expelling albendazole from the mouth in a “cloud” of dry powder. For further information on variables examined and definitions of adverse swallowing events, please see S1 Appendix.

At the end of each session (usually a full work day), additional information was recorded using a Likert scale on how often the service provider had washed his or her hands and the frequency with which a paper, spoon, or water bottle was reused between children (coded as always, most of the time, sometimes, never, or not applicable). The overall environment during drug distribution was characterized as calm, busy, or chaotic. Service providers who were available after drug distribution were interviewed regarding attitudes and practices related to deworming; awareness of the WHO recommendation to crush tablets for children 12–35 months of age; how to improve efficiency and safety of drug delivery; frequency of observed choking events in previous drug distributions; and recommended actions if a choking event occurs.

To assure inter-rater reliability, evaluators developed case definitions for adverse swallowing events (S1 Appendix) and standardized their responses before the evaluation with the help of videos. This was followed by pilot observations in Mumbai and Maharashtra, India, where two evaluators (JWK and RVD) visited distributions together and cross-checked their responses. Throughout the evaluation, all three evaluators were regularly in touch to standardize coding.

### Institutional review

The Director of Research Compliance at the University of Notre Dame formally reviewed the proposed project, determined that it did not meet the criteria for human subjects research, and classified it as program evaluation. Officials in all participating organizations and at all drug distribution sites gave consent for the evaluation. Informed consent was obtained from each service provider before being interviewed. Evaluators did not interact with or ask questions of children receiving deworming medicine or their caretakers.

### Data collection and analysis

Data were collected on a Plum Android tablet using the ODK Collect application and ONA web forms. ONA is a web-based form-builder platform that allows users to create forms to use with Open Development Kit (ODK). ODK is an open source set of tools that allow users to manage mobile data collection [25]. After data collection, the forms were uploaded to the ONA server and stored in a central location.

The data were analyzed using SPSS [26]. Bivariate associations related to adverse swallowing events were tested for statistical significance using the chi-square test, and stratified analyses were also performed to assess for confounding or interaction. Variables significantly associated with adverse swallowing events or choking were entered in multiple logistic regression models for these outcomes. Because observations might have clustered by distribution site, a variable for distribution site was included in all models. Initial models also included interaction terms for age, water, and crushed tablet form. Variables not statistically significantly associated with the outcome of interest were dropped from the model one by one in a “backwards elimination” approach to arrive at the final model.

Interviews with providers were carried out either in English or using a translator, depending on the service provider’s comfort with the English language. The interviews were audio-recorded using the Plum Android tablet or video-recorded by a Vitamin Angels team member. The interviews were then analyzed by coding pertinent recurring themes within the transcripts. The chi-square test was used to compare the frequency of responses in provider interviews and the frequency of observations on infection control and other practices.

## Results

A total of 1677 deworming treatments were observed in children 12–59 months of age. Of these, 816 (48.7%) were female. Of 65 sites in which treatments were observed, 39 were affiliated with Vitamin Angels (and were expected to follow Vitamin Angels’ drug administration and infection control protocols) and 26 were not affiliated with Vitamin Angels. The mean number of children observed per site was 25.8, with a range of 2–126.

### Drug dose

The recommended dose of albendazole increases from 200 mg (half tablet) to 400 mg (full tablet) at 24 months of age [8]. In the current evaluation, the incorrect dose of albendazole was given in 295 (17.6%) observed drug administrations. Factors associated with incorrect dosing were a busy environment (29.9% vs. 7.6%, p < 0.001) and giving uncrushed tablets (44.7% vs. 5.1%, p < 0.001). Frequency of incorrect dosing was significantly less in Vitamin Angels-affiliated sites (4.3%) than in unaffiliated sites (31.4%, p < 0.001). One NGO, not affiliated with Vitamin Angels, provided half-tablets to all children at 11 sites in Haiti, resulting in under-dosing children 2–4 years old. When this organization was excluded from the analysis, incorrect dosing was observed in 61 (4.2%) of 1442 children, 56 (91.8%) of whom were 1 or 2 years of age.

### Correct form (crushed tablets)

Among 563 children 12–35 months of age, 428 (76.0%) received the tablet in crushed form, as recommended by WHO [21]. Of 412 children treated in Vitamin Angels-affiliated sites, 411 (99.8%) received crushed tablets, compared to 17 (11.3%) of 151 children treated in sites not affiliated with Vitamin Angels (RR 8.8, p < 0.0001).

### Adverse swallowing events

Of 1677 children observed, 248 (14.8%) experienced one or more adverse swallowing events (Table 1). These 248 children experienced 458 individual adverse swallowing events, an average of 1.9 adverse swallowing events per affected child (range 1–5 events). Spitting was observed in 155 (9.2%) children, coughing in 132 (7.9%), a powder cloud in 95 (5.7%), and gagging in 48 (2.9%). Fifteen (0.9%) children experienced choking with airflow, 9 (0.5%) vomited, and 3 (0.2%) had choking in which flow of air apparently stopped for a few seconds (the child was distressed and unable to breathe or vocalize). None of the 18 choking events were fatal; the Heimlich maneuver was used in one instance. A total of 208 (12.4%) children struggled to avoid taking the tablet and 70 (4.2%) held the albendazole in their mouth without swallowing, so that ingestion could not be confirmed. Overall, 1429 (85.2%) children had no adverse swallowing event.

**Table 1:** Risk of adverse swallowing events by child-related factors, drug administration, and drug distribution setting, Haiti and India, 2017.

		Total	No. (%) with Adverse Swallowing Event	Risk Ratio (95% Confidence Interval	P-Value
Age	1 year	285	92 (32.3)	5.57 (3.86–8.04)	< 0.0001
	2 years	278	73 (26.3)	4.53 (3.10–6.64)	< 0.0001
	3 years	527	49 (9.3)	1.61 (1.05–2.45)	0.0277
	4 years	587	34 (5.8)	-	-
Gender	Male	861	155 (18.0)	1.58 (1.24–2.01)	0.0002
	Female	816	93 (11.4)	-	-
Child demeanor before delivery	Non-content	274	138 (50.4)	6.42 (5.18–7.96)	< 0.0001
	Content	1403	110 (7.8)	-	-
Child struggled during delivery	Yes	208	116 (55.8)	6.21 (5.07–7.60)	< 0.0001
	No	1469	132 (9.0)	-	-
Tablet form	Crushed	863	219 (25.4)	7.12 (4.89–10.37)	< 0.0001
	Not crushed	814	29 (3.6)	-	-
Water given	Yes	852	197 (23.1)	3.74 (2.79–5.01)	< 0.0001
	No	825	51 (6.2)	-	-
Service provider touch the child	Yes	281	34 (12.1)	0.79 (0.56–1.11)	0.1706
	No	1396	214 (15.3)	-	-
Head support	Caregiver	647	197 (30.4)	7.44 (5.27–10.50)	< 0.0001
	Healthcare provider	175	16 (9.1)	2.23 (1.26–3.94)	0.0056
	Child	855	35 (4.1)	-	-
Facility*	House	35	2 (5.7)	0.45 (0.12–1.79)	0.2601
	Other	73	11 (15.1)	1.20 (0.65–2.20)	0.5565
	Outdoors	459	107 (23.3)	1.86 (1.36–2.54)	0.0001
	School	728	80 (11.0)	0.87 (0.63–1.22)	0.4339
	Clinic	382	48 (12.6)	-	-
Environment*	Busy	772	100 (13.0)	1.44 (1.05–1.98)	0.0243
	Chaotic	327	96 (29.4)	3.26 (2.40–4.44)	< 0.0001
	Calm	578	52 (9.0)	-	-
Affiliation*	VA-affiliated	853	212 (24.9)	5.69 (4.05–7.99)	< 0.0001
	Not VA-affiliated	824	36 (4.4)	-	-

* observations taken at each site

VA–Vitamin Angels

#### Risk factors for adverse swallowing events

In bivariate analyses, several factors were associated with adverse swallowing events in aggregate, including young child age, male gender, non-content child demeanor, child struggling to resist administration of albendazole, crushed tablet form, water given, outdoor setting, chaotic or busy environment, and Vitamin Angels affiliation (Table 1). This latter association was related to the higher proportion of younger children in Vitamin Angels-affiliated sites (48.3% less than 3 years old compared to 18.3% for non-affiliated sites) and to more frequent crushing of tablets.

Risk of adverse swallowing events was highest in children 1 and 2 years of age (32.3% and 26.3%, respectively) and decreased significantly with age. Children with a non-content demeanor (i.e., combative, fussy, or fearful) *immediately before* giving albendazole were 6.4 times more likely to experience an adverse swallowing event (50.4%) than children who had a content demeanor (7.8%, p < 0.0001). Similarly, of 208 children who struggled to avoid or resist taking the albendazole *during* its administration, 116 (55.8%) experienced an adverse swallowing event, compared to 132 (9.0%) of 1469 children who did not struggle (RR 6.2, p < 0.0001). Of the 274 children who had a non-content demeanor just before albendazole administration, 173 (63.1%) struggled to avoid or resist taking the drug.

Children one year of age were more likely to have a non-content demeanor (44.9%) than children age two years (30.6%), and both groups were more likely to be non-content than three-year olds (9.1%). All three of these age groups were significantly more likely to have a non-content demeanor than children four years of age (2.2%) (p < 0.001).

Administering crushed albendazole tablets was associated with increased risk of adverse swallowing events. Among 863 children receiving crushed tablets, 219 (25.4%) experienced an adverse event, compared to 29 (3.6%) receiving a whole tablet (RR 7.1, p < 0.0001). The association between crushed tablet form and increased risk of adverse swallowing events was observed in all age groups (Table 2), with the greatest risk ratio observed in two-year-old children (RR 15.1).

**Table 2:** Number and percentage of children with adverse swallowing events, by child age and tablet form (crushed or not crushed).

		Not Crushed		Crushed		
		Total No. Children Observed	No. (%) with Adverse Swallowing Events	Total No. Children Observed	No. (%) with Adverse Swallowing Events	Risk Ratio^1^ (95% Confidence Interval)
Age	1 year	52	8 (15.4)	233	84 (36.1)	2.34* (1.21–4.53)
	2 years	83	2 (2.4)	195	71 (36.4)	15.11‡ (3.80–60.16)
	3 years	319	7 (2.2)	208	42 (20.2)	9.20‡ (4.21–20.09)
	4 years	360	12 (3.3)	227	22 (9.7)	2.91† (1.46–5.76)

‡ p-value < 0.001

† p-value < 0.01

* p-value < 0.05

^1^ Comparing crushed vs. not crushed tablets

In general, the risk of adverse swallowing events was 3.7 times greater when water was given (23.1% vs. 6.2%, p <0.0001). However, risk varied based on tablet form (crushed or whole) and whether the tablet was mixed and given together with water, water was given after the tablet, or food was given after the tablet. Compared to children receiving a whole tablet without water (4.1%), risk of adverse swallowing events was greatest (34.6%) in children receiving a crushed tablet mixed with water (RR 8.5) or when water was given immediately after the crushed tablet (29.6%, RR 7.3) (Table 3). Compared to the risk of receiving a crushed tablet without water (11.1%), risk of an adverse swallowing event remained significantly elevated for these two groups (RR 3.1 and 2.6, respectively).

**Table 3:** Risk of adverse swallowing events, by tablet form and whether and how water was given.

Administration Method	Co-Administered with:	Total No. Children Observed	No. (%) with Adverse Swallowing Events	Risk Ratio (95% Confidence Interval)
Tablet crushed	No water given	247	27 (11.1)	2.69‡ (1.58–4.60)
	Water given after	431	128 (29.6)	7.31‡ (4.78–11.21)
	Mixed with water	185	64 (34.6)	8.53‡ (5.46–13.33)
Tablet not crushed	No water given	567	23 (4.1)	-
	Water given after	236	5 (2.5)	0.52 (0.20–1.36)
	Food given after *	11	1 (9.1)	2.24 (0.33–15.15)

(‡ p-value < 0.001)

* The category “Food given after” included 11 children who received food (but not water) after being given a whole (non-crushed) tablet; in Tables 1, 4 and 5 these children were classified as not having received water.

To control for the effect of water given to children who were having trouble swallowing the albendazole or who were already experiencing an adverse swallowing event, 22 sites (38%) in which *all* children were given water were compared to the 26 sites (40%) in which *no* children were given water. The respective risk of adverse events was 23.3% and 4.6% (RR 6.2, p < 0.0001). The incidence of adverse events observed in 17 sites where some children were given water (15.1%) was significantly lower than that for sites where all children were given water and significantly higher than for sites where no children were given water. If children had a non-content demeanor, struggled to resist taking albendazole, and were given water, risk of an adverse swallowing event was 67.7%, a 17-fold increase compared to when all three factors were absent (S2 Appendix).

#### Specific adverse swallowing events

Giving crushed albendazole tablets increased risk of choking, spitting, coughing, gagging, vomiting, and producing a powder cloud; the magnitude of this risk varied by age (S3 Appendix). Giving water to children was significantly more likely to result in all three adverse swallowing events associated with a reduction in dose actually ingested: spitting (RR 3.58, p < 0.0001), vomiting (RR not calculable, p < 0.05), and producing a powder cloud (RR 5.23, p < 0.0001).

#### Adverse swallowing events, logistic regression

In a multivariate logistic regression, risk of adverse swallowing events remained significantly associated with children 1 year (OR 2.7) or 2 years (OR 2.9) of age; male gender (OR 1.6); non-content child demeanor just before albendazole administration (OR 4.3); child struggling when given albendazole (OR 2.1); and giving the child water (OR 5.8) (Table 4). The McFadden pseudo R-square value was 0.373.

**Table 4:** Factors independently associated with adverse swallowing events during administration of preventive chemotherapy for soil-transmitted helminthiasis, multivariate logistic regression.

		Odds Ratio (95% Confidence Interval)	P Value
Age	1 year	2.65 (1.49–4.72)	0.001
	2 years	2.87 (1.65–4.98)	0.000
	3 years	1.53 (0.91–2.58)	0.112
	4 years	-	
Gender	Male	1.62 (1.13–2.32)	0.009
	Female	-	
Child demeanor before drug administration	Not content	4.26 (2.60–6.99)	0.000
	Content	-	
Child struggled during drug administration	Yes	2.10 (1.24–3.54)	0.006
	No	-	
Water given	Yes	5.78 (2.38–14.01)	0.000
	No		

### Choking

Among adverse swallowing events associated with preventive chemotherapy, choking is of greatest concern. Eighteen 18 (1.1%) children experienced choking; 15 (83.3%) of these events were categorized as choke with airflow and 3 (16.7%) were categorized as choke without airflow. In bivariate analysis, factors associated with choking included tablet form, child demeanor before drug administration, child struggling during administration, whether water was given, and a chaotic drug delivery environment (Table 5).

**Table 5:** Number and percentage of choking occurrences observed by variable, at sites in Haiti and India, 2017.

		No. Children Observed	No. (%) with Choking	Risk Ratio (95% Confidence Interval)	P-Value
Age	1 year	285	4 (1.4)	2.75 (0.62–12.19)	0.184
	2 years	278	3 (1.1)	2.11 (0.43–10.40)	0.3581
	3 years	527	8 (1.5)	2.97 (0.79–11.14)	0.1064
	4 years	587	3 (0.5)	-	-
Gender	Male	861	13 (1.5)	2.46 (0.88–6.88)	0.0852
	Female	816	5 (0.6)	-	-
Child struggled during drug delivery	Yes	208	12 (5.8)	14.13 (5.36–37.23)	< 0.0001
	No	1469	6 (0.4)	-	-
Child demeanor before drug delivery	Non-content	274	15 (5.5)	25.6 (7.46–87.84)	< 0.0001
	Content	1403	3 (0.2)	-	-
Tablet form	Crushed	863	17 (2.0)	16.03 (2.14–120.22)	0.0069
	Not crushed	814	1 (0.1)	-	-
Water given	Yes	852	17 (2.0)	16.46 (2.20–123.42)	0.0064
	No	825	1 (0.1)	-	-
Service provider touched the child	No	1396	17 (1.2)	3.42 (0.46–25.61)	0.2309
	Yes	281	1 (0.4)	-	-
Head support	Caregiver	647	17 (2.6)	nc	nc
	Healthcare provider	175	1 (0.6)	nc	nc
	Child (self)	856	0 (0)	-	-
Facility*	House	35	0 (0)	1.52 (0.08–28.85)	0.7804
	Other	73	2 (2.7)	3.49 (0.59–20.51)	0.2669
	Outdoors	459	8 (1.7)	2.22 (0.59–8.31)	0.2365
	School	728	5 (0.7)	0.87 (0.21–3.64)	0.8538
	Clinic	382	3 (0.8)	-	-
Environment*	Busy	772	4 (0.5)	1 (0.22–4.44)	0.9982
	Chaotic	327	11 (3.4)	6.48 (1.82–23.06)	0.0039
	Calm	578	3 (0.5)	-	-

* data collected on each site rather than for each child

nc–not calculable

Of the 863 children who received a crushed albendazole tablet, 17 (2.0%) experienced choking, compared to 1 (0.1%) of 814 children who were given non-crushed tablets (RR 16.0, p = 0.007). Compared to children receiving a whole tablet without water, those who received a crushed tablet with water had a significantly increased risk of choking, regardless of whether the tablet was mixed with water (4 [2.2%] of 185 children) or given afterward (13 [3.0%] of 431 children). No children receiving a crushed tablet without water had a choking episode. Only one (0.1%) child who received a tablet that was not crushed had a choking episode, and that child did not receive water.

In the 133 children for whom all three risk factors–non-content child demeanor before administration, child struggling during administration, and the addition of water–were present, 12 (9.0%) experienced choking, compared to three (0.2%) of 1368 children with a content demeanor who did not struggle, a 41-fold increase. None of the 729 children who were content, did not struggle, and were not offered water experienced choking.

Four instances were observed of a child’s nose being held to force them to swallow the tablet. All four cases involved a crushed tablet and three (75%) resulted in the child choking. In these three instances, the child struggled to avoid taking the tablet. The child whose nose was held but did not choke did not struggle or try to avoid taking the tablet. Providers responded to choking incidents by repositioning the child and, in one case, by administering the Heimlich maneuver.

In a multiple logistic regression model, when adjusting for potential clustering by site, the only factor remaining independently associated with choking was non-content child demeanor before drug administration (OR 20.6, CI 5.4–78.0). The McFadden pseudo R-square value was 0.44.

### Infection prevention

Service providers in Vitamin Angels-affiliated sites touched children much less frequently (5.5% of child contacts) than did providers in sites not affiliated with Vitamin Angels (28.4%), and they were more likely to always wash their hands between children (46.2% vs. none). Twenty-three (88.5%) of 26 Vitamin Angels-affiliated sites disposed of the paper used to crush the tablet after each use, compared to 3 (100%) of 3 non-affiliated sites. Service providers in 11 sites used spoons to give albendazole tablet. In the 9 such sites affiliated with Vitamin Angels, spoons were always washed with soap and water before reuse, while in the two non-affiliated sites, the spoons were never washed before reuse. In contrast, of 33 sites that used containers (i.e., cups or bottles) to give children water with or immediately after albendazole, the containers were always washed between children in 1 (5.3%) of 19 Vitamin Angels-affiliated sites and in 3 (21.4%) of 14 sites not affiliated with Vitamin Angels.

### Service provider interviews

A total of 29 service providers were interviewed. Of those, 17 were from sites using the Vitamin Angels approach and 12 were from sites not using the Vitamin Angels approach. None of 12 service providers in sites not using the Vitamin Angels approach were aware that WHO recommends crushing the tablets for children under three years old. (Service providers in Vitamin Angels-affiliated sites already practiced crushing albendazole tablets and were not asked this question). When asked about the best method to crush a tablet, most providers indicated that folding the tablet in a clean piece of paper and crushing it with a heavy bottle was their preferred method. Three providers preferred a mechanical pill crusher, and one found a stone to be an acceptable tool.

Of 23 service providers who were asked, 19 (82.6%) reported knowing, in general, what to do to clear the airway if a child chokes (85.7% of 14 providers and 77.8% of 9 providers affiliated and not affiliated with Vitamin Angels, respectively). For example, they knew that very small children should be placed face-down and struck on the back. Service providers were not asked to demonstrate proficiency. Most were not familiar with the term “Heimlich maneuver.”

Three of the service providers interviewed reported previously seeing a child choke during deworming. They said that the child choked because he or she was not held in the proper position. In two of these instances, service providers had given the child water to alleviate the choking and in the other, the provider performed the Heimlich maneuver.

Service providers recommended that parents be told how to position the child’s head during distribution, which could help prevent choking and other adverse swallowing events. Another common recommendation was to provide a liquid formulation of deworming medication.

## Discussion

Deaths in young children caused by choking on medicine are preventable. In high-income countries, age-appropriate pediatric formulations of many medicines are available, which reduce choking risk and facilitate acceptance [27,28]. Such formulations of albendazole and mebendazole are not widely available in countries where WHO recommends preventive chemotherapy for STH.

In the current evaluation, 18 (1.1%) of 1677 children who were given albendazole for STH experienced choking, 15 with airflow and 3 in which airflow appeared to be transiently interrupted. This rate is similar to the incidence observed in Madagascar, where choking associated with deworming occurred in 1% of children 12–24 months of age and 3% of children 25–36 months of age. A lower risk of choking (0.1%) during deworming was observed in Rwanda [21]. If one assumes the overall risk of choking to be ~1% for children 1–4 years of age, with roughly 150 million children of this age being dewormed annually [9], one could reasonably expect 1.5 million of them to experience choking.

### Factor associated with choking–child demeanor

In the multivariate analysis, only one risk factor remained significantly associated with choking: non-content demeanor of the child just before giving albendazole, with an odds ratio of 20.6. A multitude of influences probably affect child demeanor, including the environmental setting, attitude of the service provider, relationship between child and caregiver, how the albendazole is delivered, and other factors associated with child health and well-being.

Child demeanor is a modifiable risk factor. In some of the drug delivery sites, service providers asked caregivers to step out of line for a few moments to calm their child, after which albendazole was administered without incident. Our data suggest that by calming fussy, fearful, or combative children before albendazole administration, risk of choking could be reduced from the observed 1.1% to 0.2% (Table 5). However, this may take additional time–a rare commodity in busy child health day settings–as well as patience and skill on the part of service providers and caregivers. In a recent paper describing the safety and efficacy of a new formulation of mebendazole for young children, Friedman et al. reported very few adverse swallowing events [29]. They emphasized, however, that “great care was taken in collaboration with their caregiver (mainly mothers) to convince the child to chew and swallow the tablets. The children were never forced to chew or swallow the tablets, though, in some instances, a substantial length of time was required to complete this process.”

### Factors associated with adverse swallowing events

Although choking was the outcome of greatest concern in this evaluation, we also monitored the frequency of other adverse swallowing events potentially associated with choking, such as coughing and gagging, as well as those associated with reduced ingestion of albendazole, including spitting, vomiting, and expulsion of the finely crushed tablet in a powder cloud. While of lesser concern than choking, inadequate dosing may be a concern at the population level, as sub-therapeutic doses can potentially contribute to the development of drug resistance [30].

In multivariate analysis, several factors were independently associated with increased risk of adverse swallowing events, including non-content child demeanor–also an independent predictor of choking, noted above–as well as male gender, child age of 1 or 2 years; giving the child water; and child struggling when given the albendazole (Table 4).

#### Child age

Choking and other adverse swallowing events are more common in children less than three years old for developmental and anatomic reasons. In our evaluation, children less than three years of age were significantly more likely to have a non-content demeanor and to struggle during albendazole administration, suggesting that age-related attitudes and behaviors contribute to the increased risk in this age group.

#### Water

The observation that giving water increased risk of adverse swallowing events was unexpected. When someone is observed coughing or struggling with irritation of the airway, it is natural to offer them a glass of water. It is likely that water *per se* is not the risk factor, but rather, the way it was given. In order to avoid contaminating cups, or because clean cups were not available, water was often poured into the mouths of the children to avoid the water vessel touching the child’s lips. This required the child’s neck to be hyperextended. At other times, water was given to children in their own cups and they drank the water without help. Systemic records were not kept on how water was given during this evaluation. However, our incidental observations, which warrant further assessment, suggest that risk associated with water could be reduced by avoiding hyperextension of the neck.

#### Child struggled to avoid taking albendazole

The independent association between adverse swallowing events and both a non-content demeanor *before* giving albendazole and a child struggling *during* drug administration suggests that adverse swallowing events can be reduced by paying attention to the child at these two specific time points and delaying (or even cancelling) administration if necessary. Excluding the 309 children who had a fussy, fearful, or combative demeanor or who struggled against taking albendazole would have reduced risk of adverse swallowing events from 14.8% to 6.9%. The “cost” of this prevention would have been a reduction in drug coverage by 18.4% (S2 Appendix). However, 126 (40.8%) of these children experienced an adverse swallowing event that, to some degree, reduced the dose of drug ingested (i.e., spitting, vomiting, or powder cloud), and an additional 7 (2.3%) of them refused to swallow the medicine. Thus, overall, non-content demeanor and struggling to avoid taking albendazole resulted in 133 (43.0%) of these children receiving less than the recommended dose; these losses could be avoided through efforts to engage the child’s cooperation.

#### Crushed tablet

Although crushed tablets were associated with both choking and adverse swallowing events in bivariate analyses, these associations were not statistically significant in the multivariate analysis. The potential association, however, warrants a brief comment. Two factors might explain such an association, if it exists. First, aspiration of powder is more likely when the tablet is crushed; crushed tablets increased risk of both cough and production of a powder cloud (S3 Appendix). Second, as with water, pouring the powder from a folded paper into a child’s mouth may require extension of the neck, which opens the trachea and predisposes to adverse swallowing events. This can be avoided if a child self-administers a whole tablet and chews it. The angle of the neck (i.e., extension or flexion) was not systematically assessed in this evaluation. However, in bivariate analysis, a child who could support his or her own head had a significantly lower risk of an adverse swallowing event (4.1%) than a (generally younger) child whose head was supported by a service provider (9.1%) or the child’s caregiver (30.4%) (Table 1). Anecdotally, when the child’s head was supported by his or her caregiver (usually the parent), the neck was often hyperextended.

### WHO recommendations and implications for prevention

To reduce the risk of fatal choking with preventive chemotherapy for STH, WHO recommends: 1) crushing deworming tablets for children <3 years of age; 2) using two spoons to crush the tablets; 3) giving crushed tablets with water; 4) not forcing treatment on children who resist; and 5) training service providers what to do if a child chokes. The most detailed WHO recommendations, which appeared in a 2007 PPC newsletter, [21] have not received adequate attention, perhaps because they were not published as official WHO guidelines.

#### Crushing tablets for children less than 3 years of age

In multivariate analyses, crushed tablets were not significantly associated with choking or adverse swallowing events in general. Based on the scant information available, fatal cases of choking during deworming young children have involved whole tablets or tablets that were not crushed [17]. General reviews of data on childhood choking indicate that solid, round, cylindrical or ovoid objects that can conform to the shape of the trachea are most likely to completely occlude the airway [31,32]. Therefore, despite the paucity of data, crushing tablets for children 1–2 years of age probably decreases risk of fatal choking. It is concerning that in sites not affiliated with Vitamin Angels, only 11% of children 1–2 years of age received crushed tablets.

It remains an open question whether deworming tablets should be crushed for children 3–4 years of age. In the current evaluation, adverse swallowing events and choking tended to be higher in in children 1–2 years of age, although 1.5% of 3-year-old children had a choking event. A policy of “zero tolerance” for choking deaths would favor extending the WHO recommendation of crushing tablets to include children 3 years of age. Data from the current evaluation suggest that choking risk decreases markedly in children 4 years of age. However, procedural consistency and logistical concerns might favor a policy of crushing deworming tablets for all preschool-age children in some settings.

#### Using spoons to crush tablets

WHO suggests that deworming tablets be crushed between two spoons [20,21]. Several factors make this practice difficult in the field, including the hardness of some albendazole tablets. Vitamin Angels recommends the “paper and bottle” method initially suggested by the WHO focal point for STH control, Dr. Antonio Montresor (A. Montresor, personal communication, 2015). In the current evaluation, virtually all service providers who use this approach found it feasible and acceptable. However, evaluators observed that the noise made by smashing the tablet with a heavy bottle sometimes startled young children. If possible, tablets should be crushed away from where albendazole is being given.

#### Giving tablets with water

Unexpectedly, it was found that giving water increased risk of adverse swallowing events, even when controlling for other factors in the regression analysis. As noted above, our incidental observations suggest that this increased risk may have been associated with the practice of pouring the water into the child’s mouth in an effort to avoid contamination of the water vessel. Our findings do not argue against the common practice of providing water to children who are having difficulty swallowing, especially if they can hold the container (with or without assistance) and control the flow.

#### Not forcing the child to take the drug

In the PPC newsletter [21], WHO cautions not to “hold the child’s nose to make him or her swallow,” and never to force a child to take albendazole. Our findings strongly support this recommendation. The evaluators observed four instances where the child’s nose was held to “encourage” him or her to swallow the albendazole, three of whom choked. The PPC newsletter states, “if administration is unsuccessful, *do* let the child go home untreated… he/she will be treated during the next round.” Our findings indicate that drug coverage would only be reduced by 18.4% by not forcing children to take albendazole who are fussy, fearful, or combative, or who struggle to resist it.

#### Heimlich maneuver

In the 2007 PPC newsletter [21], WHO recommended that “staff administering tablets to small children should be trained in what do to if a child chokes,” and provided figure drawings of the Heimlich maneuver. It is unclear the extent to which STH program managers are aware of this recommendation or the extent to which service providers have been trained. Arguments against requiring service providers to demonstrate competency in the Heimlich maneuver have centered on the cost of such training and the perceived rarity of choking events. In the current evaluation, 83% of service providers reported knowing, in general, what to do to clear the airway if a child chokes, suggesting that they already have some familiarity with the Heimlich maneuver (even if they do not know it by that name). Refresher training could save lives both during STH preventive chemotherapy and in other community settings.

### Infection prevention

Within the neglected tropical disease (NTD) community, little attention is given to the potential for person-to-person or fomite transmission of gastrointestinal and respiratory pathogens during mass drug administration. Infectious disease outbreaks have seldom, if ever, been reported in association with mass drug administration, although this is not surprising since surveillance and reporting of infectious disease outbreaks is limited in such settings. Vitamin Angels recommends simple infection control policies to reduce the potential for nosocomial transmission. Our observations indicate that service providers in Vitamin Angels-affiliated sites were less likely to touch children during albendazole administration and much more likely to wash their hands between children than providers in sites not affiliated with Vitamin Angels. These differences may be attributed to training programs that Vitamin Angels provides to its partner NGOs, as well as their use of visual checklists [23] to remind providers of recommended practices. However, even in Vitamin Angels-affiliated sites, there is room for improvement. For example, as noted, only 1 (3.8%) of 19 Vitamin Angels-affiliated sites that used cups or water bottles always washed them between children. Sharing water vessels puts children at risk of infectious disease transmission. If there is no option other than to share water bottles or cups, the practice of providing water with each albendazole tablet should be reconsidered, since it is also associated with increased risk of adverse swallowing events.

### Limitations

The evaluation had several limitations. Despite efforts to standardize the coding and recording of adverse swallowing events, some events may have been misclassified in the busy, and sometimes chaotic, settings in which children were dewormed. Selection of evaluation sites was determined by drug distribution schedules, relative proximity to other distributions, and knowledge of Vitamin Angels staff of ongoing deworming activities in both Vitamin Angels-affiliated and non-affiliated sites during a 6-week period. Therefore, it is unclear the degree to which our findings are representative of other sites, even within India and Haiti.

Further, it is likely that service providers and caregivers altered their behavior in the presence of the evaluator. Evaluators (JWK, RVD, and AMM) were graduate students at the University of Notre Dame, not Vitamin Angels employees. They received training in the theoretical and practical aspects of preventive chemotherapy before traveling to Haiti and India. However, they were often accompanied by representatives of Vitamin Angels. In addition, interviews with service providers often required translation into one or two languages. In some cases, the service providers’ supervisors helped to translate. This may have led to some responses that were socially desirable but not factually accurate. Finally, sample size was inadequate to assess risk factors for certain outcomes, and there may be confounding variables that were not accurately accounted for or measured.

### Conclusions

Despite these limitations, this evaluation provides a unique “snapshot” of deworming practices in settings where deworming and vitamin A supplementation programs are implemented. Assessments of drug safety for preventive chemotherapy are usually done in carefully-monitored research studies, focusing on pharmacologic reactions and adverse medical events that occur hours or days after administration [15,29,33]. In contrast, this evaluation provides information on practices and experiences during deworming in program settings. It assessed risk of adverse swallowing events and established a baseline against which modified practices or new drug formulations might be measured.

A new disintegrating formulation of mebendazole has been developed by Johnson & Johnson and is underdoing prequalification by WHO [33]. Preliminary data suggest that this formulation may be ideal for preschool-age children. However, its safety in terms of aspiration and choking has not yet been tested in large-scale trials under field conditions. Future studies should evaluate the feasibility and safety of this and other formations of drugs for preventive chemotherapy. Additional work is also needed to improve awareness of current practices, skills, and knowledge of service providers and to better understand the role of other factors, such as the angle of the neck during drug administration, in contributing to adverse swallowing events.

## Supporting information

S1 Checklist STROBE checklist for cross-sectional studies. (DOC)

S1 Appendix Variable definitions and potential values, including case definitions for adverse swallowing events, India and Haiti, 2017. (DOCX)

S2 Appendix Effect of child demeanor immediately before albendazole administration, child struggling to resist taking albendazole, and giving the child water on risk of adverse swallowing events during preventive chemotherapy for soil-transmitted helminthiasis, India and Haiti, 2017. (DOCX)

S3 Appendix Frequency of adverse swallowing events (ASEs) by age and tablet form (i.e., crushed or not) during preventive chemotherapy for soil-transmitted helminthiasis, India and Haiti, 2017. (DOCX)
