# Supplementary material for: Risk of adverse swallowing events and choking during deworming for preschool-aged children
Source: PLoS Negl Trop Dis. 2018 Jun 22;12(6):e0006578. doi: 10.1371/journal.pntd.0006578 (PMC6014639; doi:10.1371/journal.pntd.0006578)
Supplement: S1 Checklist — (DOC) [file pntd.0006578.s001.doc]

**S1 Checklist. STROBE Checklist for cross-sectional studies**

**Manuscript:** Kernell et al. Risk of adverse swallowing events and choking during deworming for preschool-aged children

**Note to the Editors: This was not a “study” but rather, a limited cross-sectional *evaluation* of programs that provide deworming tablets to preschool-age children for control of soil-transmitted helminthiasis.**

|  | Item No | Recommendation |
| --- | --- | --- |
| **Title and abstract** | 1 | (*a*) Indicate the study’s design with a commonly used term in the title or the abstract. **Study design was observation. We noted in the abstract (line 34) that albendazole distributions were *observed*.** |
|  |  | (*b*) Provide in the abstract an informative and balanced summary of what was done and what was found. **We focused on the major outcomes in lines 35-47.** |
| Introduction | | |
| Background/rationale | 2 | Explain the scientific background and rationale for the investigation being reported. **See Introduction, lines 87-121.** |
| Objectives | 3 | State specific objectives, including any prespecified hypotheses. **Three primary objectives are listed in the last paragraph of the Introduction, lines 134-142. Objective 3 indicates several hypotheses: that the Vitamin Angels approach is associated 1) with greater likelihood of giving the correct dose and form of albendazole; 2) with decreased risk of adverse swallowing events; and 3) with improved infection control practices (lines 138-142).** |
| Methods | | |
| Study design | 4 | Present key elements of study design early in the paper. **In the abstract (line 34) and introduction (line 134) the fundamental element of the study design (“observation”) is highlighted. Additional details are provided in the Methods section, in lines 152-173**. |
| Setting | 5 | Describe the setting, locations, and relevant dates, including periods of recruitment, exposure, follow-up, and data collection**. Setting and locations: lines 144-151. Periods of recruitment: line 146. Exposure: N/A. Follow-up: none. Data collection: lines 152-173.** |
| Participants | 6 | (*a*) Give the eligibility criteria, and the sources and methods of selection of participants. **Provided in lines 134 and 144-151**. |
| Variables | 7 | Clearly define all outcomes, exposures, predictors, potential confounders, and effect modifiers. **Outcomes: adverse swallowing events are detailed in lines 160-163 and defined in more detail in S1 Appendix. Infection control outcomes: lines 165-168. Knowledge/attitudes “outcomes”: lines 169-173. Exposure: child given albendazole (line 1463).** **Potential confounders and effect modifiers: demographics (line 155-6); demeanor (line 156), struggling (157), and facility characteristics (168-169).** Give diagnostic criteria, if applicable. **Not strictly applicable, but see S1 Appendix.** |
| Data sources/ measurement | 8* | For each variable of interest, give sources of data and details of methods of assessment (measurement). Describe comparability of assessment methods if there is more than one group**. All data on adverse swallowing events were collected by observing albendazole distribution (lines 154 and following). Data on other variables were collected by observing the facility (lines 152-154) or the provider (lines 165-168), or by interviewing the provider (lines 166-173).** |
| Bias | 9 | Describe any efforts to address potential sources of bias. **Lines 174-179.** |
| Study size | 10 | Explain how the study size was arrived at. **As this was an initial descriptive evaluation, calculating a sample size required to test a single overriding hypothesis was not a useful or meaningful academic exercise. Therefore, we did not record it in the manuscript. However, we hypothesized a 5% difference of adverse event frequency between the Vitamin Angels approach and the WHO approach, requiring at least 380 children 12-59 months old, given an alpha of 0.05 (based on a 1-sided hypothesis), power of 80%, and non-response of 10%.** |
| Quantitative variables | 11 | Explain how quantitative variables were handled in the analyses. **See lines 193-201**. If applicable, describe which groupings were chosen and why. N/A |
| Statistical methods | 12 | (*a*) Describe all statistical methods, including those used to control for confounding. **See lines 193-201**. |
|  |  | (*b*) Describe any methods used to examine subgroups and interactions. **Lines 193-201.** |
|  |  | (*c*) Explain how missing data were addressed. **Variables of interest did not have missing data.** |
|  |  | (*d*) If applicable, describe analytical methods taking account of sampling strategy. N/A |
|  |  | (*e*) Describe any sensitivity analyses. N/A |
| Results | | |
| Participants | 13* | (a) Report numbers of individuals at each stage of study—eg numbers potentially eligible, examined for eligibility, confirmed eligible, included in the study, completing follow-up, and analysed. **Number of children observed: line 209. Number of service providers interviewed: line 341.** |
|  |  | (b) Give reasons for non-participation at each stage. N/A |
|  |  | (c) Consider use of a flow diagram. **Not necessary for cross-sectional study.** |
| Descriptive data | 14* | (a) Give characteristics of study participants (eg demographic, clinical, social) and information on exposures and potential confounders. **Characteristics: lines 209-213 and Table 1. Information on confounders or risk factors is provided throughout the results as well as in Tables 1, 2, 3, and 5.** |
|  |  | (b) Indicate number of participants with missing data for each variable of interest. N/A |
| Outcome data | 15* | Report numbers of outcome events or summary measures. **Lines 231-241**. |
| Main results | 16 | (*a*) Give unadjusted estimates and, if applicable, confounder-adjusted estimates and their precision (eg, 95% confidence interval). Make clear which confounders were adjusted for and why they were included. **Unadjusted estimates in Tables 1,2,3,5. Adjusted estimates Table 4, lines 293-297, and lines 324-327.** |
|  |  | (*b*) Report category boundaries when continuous variables were categorized. See above. N/A |
|  |  | (*c*) If relevant, consider translating estimates of relative risk into absolute risk for a meaningful time period. N/A |
| Other analyses | 17 | Report other analyses done—eg analyses of subgroups and interactions, and sensitivity analyses. **Tables 2 and 3 and Appendices S2 and S3.** |
| Discussion | | |
| Key results | 18 | Summarise key results with reference to study objectives. **Lines 369-371, 402-405; throughout discussion.** |
| Limitations | 19 | Discuss limitations of the study, taking into account sources of potential bias or imprecision. Discuss both direction and magnitude of any potential bias. **Lines 524-541.** |
| Interpretation | 20 | Give a cautious overall interpretation of results considering objectives, limitations, multiplicity of analyses, results from similar studies, and other relevant evidence. **Lines 364-523**. |
| Generalisability | 21 | Discuss the generalisability (external validity) of the study results. **Lines 525-531**. |
| Other information | | |
| Funding | 22 | Give the source of funding and the role of the funders for the present study and, if applicable, for the original study on which the present article is based. **Provided in financial disclosure.** |

*Give information separately for exposed and unexposed groups.

.

**Note:** An Explanation and Elaboration article discusses each checklist item and gives methodological background and published examples of transparent reporting. The STROBE checklist is best used in conjunction with this article (freely available on the Web sites of PLoS Medicine at http://www.plosmedicine.org/, Annals of Internal Medicine at http://www.annals.org/, and Epidemiology at http://www.epidem.com/). Information on the STROBE Initiative is available at www.strobe-statement.org.
