# Supplementary material for: Risk of adverse swallowing events and choking during deworming for preschool-aged children
Source: PLoS Negl Trop Dis. 2018 Jun 22;12(6):e0006578. doi: 10.1371/journal.pntd.0006578 (PMC6014639; doi:10.1371/journal.pntd.0006578)
Supplement: S1 Appendix — (DOCX) [file pntd.0006578.s002.docx]

**S1 Appendix. Variable definitions and potential values, including case definitions for adverse swallowing events, India and Haiti, 2017**

Observations taken at each drug distribution site:

1. Distribution site name
2. Field partner name
3. Location [collected through GPS]
4. Country
   1. India
   2. Haiti
5. Time drug distribution started
6. Organization affiliation
   1. Vitamin Angels affiliate
   2. Not affiliated with Vitamin Angels
7. Deworming facility
   1. Clinic
   2. Outdoors
   3. House
   4. School
   5. Hospital
   6. House-to-house
   7. Other (specify)
8. What supplies are found at the site?
   1. Vitamin Angels drug distribution visual checklist
   2. Deworming tablets
   3. Alcohol-based hand sanitizer
   4. Soap
   5. Water
   6. Small, clean, white paper
   7. Bottle to crush tablet
   8. Plastic bag for trash
   9. Pens
   10. Record keeping tool (i.e. tally sheet or distribution register)
   11. Scissors
9. Time drug distribution ended
10. Number of providers observed
11. Number of children treated
12. Environment/setting
    1. Calm, relaxed
    2. Busy but not chaotic
    3. Chaotic, crowded, noisy
13. Are tablets crushed before the child is seen?
    1. Tablets crushed before the child is seen
    2. Tablets crushed as child is seen
    3. Tablets never crushed
14. Is water provided to each child?
    1. All children
    2. Some children
    3. No children
15. What type of water is provided?
    1. No water
    2. Untreated water
    3. Bottled water
    4. Treated water – boiled
    5. Treated water – chlorinated
    6. Treated water – filtered
    7. Other (specify)
16. Was the paper used to crush the tablets disposed of after each use?
    1. Always
    2. Most of the time
    3. Sometimes
    4. Never
    5. N/A
17. Service provider identification number
18. Did the service provider wash his/her hands between children?
    1. Always
    2. Most of the time
    3. Sometimes
    4. Never
19. Did the service provider touch the children?
    1. Always
    2. Most of the time
    3. Sometimes
    4. Never
    5. N/A
20. Additional comments

Observations taken for each albendazole administration:

1. Child age (record one)
   1. In months
   2. In years
   3. Birthdate
2. Gender
   1. Male
   2. Female
3. Dose received
   1. Half (200 mg)
   2. Whole (400 mg)
   3. Other (specify)
4. Was the tablet crushed?
   1. Tablet not crushed
   2. Crushed between two spoons
   3. Crushed by bottle in paper
   4. Crushed by a mortar and pestle
   5. Crushed by another method
5. Child demeanor
   1. Combative
   2. Fussy or Fearful
   3. Content
   4. Other
6. Who supports the child’s head?
   1. Caregiver
   2. Service or healthcare provider
   3. Child
7. Does the service provider touch the child?
   1. Yes
   2. No
8. How is the tablet administered? (check all that apply)
   1. Tablet given (not crushed)
   2. Crushed tablet poured into mouth – no water
   3. Crushed tablet mixed with water
   4. Crushed tablet mixed with food
   5. Water given after dose
   6. Food given after dose
   7. Other (specify)
9. Child tolerance/reaction to albendazole (see also adverse swallowing event definitions, below):
   1. No reaction, took without incident
   2. Struggled, tried to avoid taking
   3. Choke (with airflow)
   4. Choke (airflow stopped)
   5. Spit
   6. Cough
   7. Gag
   8. Vomit
   9. Powder cloud
   10. Tablet held in mouth
10. Did the provider child to see if the child was calm post-albendazole administration?
    1. Yes
    2. No

**Adverse swallowing event case definitions:**

An adverse swallowing event was recorded when any of the following were observed upon administration of albendazole:

1. Choke (airflow stopped): Child is visibly distressed, is unable to breathe and unable to vocalize.
2. Choke (with airflow): Child is visibly distressed, but able to vocalize; coughs intensely and struggles to eject material from trachea.
3. Spit: Child expels moistened tablet or tablet fragments from mouth, with or without a “spitting” sound.
4. Cough: Child forcefully expels air from lungs with a sudden, sharp sound.
5. Gag: Child leans head forward and sticks out tongue, may or may not make a gagging noise (see <https://www.youtube.com/watch?v=EcJ23l-23Qc>).
6. Vomit: Child expels contents of stomach in addition to powder or tablet.
7. Powder cloud: Child expels dry powder (from crushed tablet) from mouth in a “cloud” that may or may not leave powder on the face. (For a highly exaggerated example of powder cloud unrelated to deworming, see <https://www.youtube.com/watch?v=-9dHiTwp4Z4>)
