# Supplementary material for: Risk of adverse swallowing events and choking during deworming for preschool-aged children
Source: PLoS Negl Trop Dis. 2018 Jun 22;12(6):e0006578. doi: 10.1371/journal.pntd.0006578 (PMC6014639; doi:10.1371/journal.pntd.0006578)
Supplement: S2 Appendix — (DOCX) [file pntd.0006578.s003.docx]

**S2 Appendix**. Effect of child demeanor immediately before albendazole administration, child struggling to resist taking albendazole, and giving the child water on risk of adverse swallowing events during preventive chemotherapy for soil-transmitted helminthiasis, selected sites in India and Haiti, 2017

| Child Demeanor | Child Struggled | Water Given | Total | No. (%) with Adverse Swallowing Event | Risk Ratio^1^ | Risk Ratio^2^ | Risk Ratio^3^ | Risk Ratio^4^ |
| --- | --- | --- | --- | --- | --- | --- | --- | --- |
| Not content | Yes | Yes | 133 | 90 (67.7) | 17.01‡ | 2.71‡ | 1.54† | 6.42‡ |
|  |  | No | 40 | 10 (25.0) | 6.28‡ | - |  |  |
|  | No | Yes | 59 | 30 (50.8) | 12.78‡ | 2.67† | - |  |
|  |  | No | 42 | 8 (19.0) | 4.79‡ | - |  |  |
| Content | Yes | Yes | 21 | 12 (57.1) | 14.36‡ | 2.00 | 6.65‡ | - |
|  |  | No | 14 | 4 (28.6) | 7.18‡ | - |  |  |
|  | No | Yes | 639 | 65 (10.2) | 2.56‡ | 2.56‡ | - |  |
|  |  | No | 729 | 29 (4.0) | - | - |  |  |

(‡ p-value < 0.001, † p-value < 0.01)

1 – Comparison group: content children who did not struggle and did not receive water

2 – Comparison group: children who did not receive water within each category of demeanor and struggle

3 – Comparison group: children who did not struggle within categories of child demeanor (content and non-content)

4 – Comparison group: all children with content demeanor
