# Supplementary material for: Risk of adverse swallowing events and choking during deworming for preschool-aged children
Source: PLoS Negl Trop Dis. 2018 Jun 22;12(6):e0006578. doi: 10.1371/journal.pntd.0006578 (PMC6014639; doi:10.1371/journal.pntd.0006578)
Supplement: S3 Appendix — (DOCX) [file pntd.0006578.s004.docx]

**S3 - Appendix**. Frequency of adverse swallowing events (ASEs) by age and tablet form (i.e., crushed or not) during preventive chemotherapy for soil-transmitted helminthiasis, India and Haiti, 2017

|  | Tablet  Form | Age in Years | | | | |
| --- | --- | --- | --- | --- | --- | --- |
|  |  | 1 | 2 | 3 | 4 | Total |
| Number of children observed | Crushed | 233 | 195 | 208 | 227 | 863 |
|  | Not crushed | 52 | 83 | 319 | 360 | 814 |
| No. (%) with ASE | Crushed | 84 (36.1)* | 71 (36.4)‡ | 42 (20.2)‡ | 22 (9.7)† | 219 (25.4)‡ |
|  | Not crushed | 8 (15.4)* | 2 (2.4)‡ | 7 (2.2)‡ | 12 (3.3)† | 29 (3.6)‡ |
| No. (%) with no ASE | Crushed | 149 (63.9) | 124 (63.6) | 166 (79.8) | 205 (90.3) | 644 (74.6)‡ |
|  | Not crushed | 44 (84.6) | 81 (97.6) | 312 (97.8) | 348 (96.7) | 785 (96.4)‡ |
| No. (%) choked with airflow | Crushed | 3 (1.3) | 3 (1.5)* | 6 (2.9)* | 2 (0.9) | 14 (1.6)* |
|  | Not crushed | 0 (0) | 0 (0)* | 1 (0.3)* | 0 (0) | 1 (0.1)* |
| No. (%) choked without airflow | Crushed | 1 (0.4) | 0 (0) | 1 (0.5) | 1 (0.4) | 3 (0.3) |
|  | Not crushed | 0 (0) | 0 (0) | 0 (0) | 0 (0) | 0 |
| No. (%) spitting | Crushed | 38 (16.3) | 54 (27.7)† | 28 (13.5)‡ | 12 (5.2) | 132 (15.3)‡ |
|  | Not crushed | 8 (15.4) | 1 (1.2)† | 6 (1.9)‡ | 8 (2.2) | 23 (2.8)‡ |
| No. (%) with cough | Crushed | 50 (21.5)* | 42 (21.5)† | 24 (11.5)‡ | 12 (5.3)† | 128 (14.8)‡ |
|  | Not crushed | 0 (0)* | 1 (1.2)† | 2 (0.6)‡ | 1 (0.3)† | 4 (0.5)‡ |
| No. (%) with gag | Crushed | 12 (5.2) | 17 (8.7) | 7 (3.4)* | 9 (4.0)* | 45 (5.2)‡ |
|  | Not crushed | 0 (0) | 0 (0) | 1 (0.3)* | 2 (0.6)* | 3 (0.4)‡ |
| No. (%) vomiting | Crushed | 3 (1.3) | 3 (1.5) | 1 (0.5) | 2 (0.9) | 9 (1.0)* |
|  | Not crushed | 0 (0) | 0 (0) | 0 (0) | 0 (0) | 0* |
| No. (%) with powder cloud | Crushed | 37 (15.9)* | 36 (18.5)* | 15 (7.2) | 7 (3.1)* | 95 (11.4)‡ |
|  | Not crushed | 0 (0)* | 0 (0)* | 0 (0) | 1 (0.3)* | 1 (0.1)‡ |

(‡ p-value < 0.001, † p-value < 0.01, * p-value < 0.05)
